# Antifreeze glycopeptides: from structure and activity studies to current approaches in chemical synthesis

**DOI:** 10.1007/s00726-016-2368-z

**Published:** 2016-12-02

**Authors:** Małgorzata Urbańczyk, Jerzy Góra, Rafał Latajka, Norbert Sewald

**Affiliations:** 1 Department of Organic and Pharmaceutical Technology, Faculty of Chemistry, Wrocław University of Science and Technology, Wybrzeże St. Wyspiańskiego 29, 50-370 Wrocław, Poland; 2 Organic Chemistry III, Department of Chemistry, Bielefeld University, Universitätsstrasse 25, 33615 Bielefeld, Germany

**Keywords:** Antifreeze glycopeptides, AFGP, Structure–activity relationship, Solid-phase peptide synthesis, Hydration shell dynamics, Terahertz spectroscopy

## Abstract

Antifreeze glycopeptides (AFGPs) are a class of biological antifreeze agents found predominantly in Arctic and Antarctic species of fish. They possess the ability to regulate ice nucleation and ice crystal growth, thus creating viable life conditions at temperatures below the freezing point of body fluids. AFGPs usually consist of 4–55 repetitions of the tripeptide unit Ala–Ala–Thr that is *O*-glycosylated at the threonine side chains with β-d-galactosyl-(1 → 3)-α-*N*-acetyl-d-galactosamine. Due to their interesting properties and high antifreeze activity, they have many potential applications, e.g., in food industry and medicine. Current research is focused towards understanding the relationship between the structural preferences and the activity of the AFGPs, as well as developing time and cost efficient ways of synthesis of this class of molecules. Recent computational studies in conjunction with experimental results from NMR and THz spectroscopies were a possible breakthrough in understanding the mechanism of action of AFGPs. At the moment, as a result of these findings, the focus of research is shifted towards the analysis of behaviour of the hydration shell around AFGPs and the impact of water-dynamics retardation caused by AFGPs on ice crystal growth. In the field of organic synthesis of AFGP analogues, most of the novel protocols are centered around solid-phase peptide synthesis and multiple efforts are made to optimize this approach. In this review, we present the current state of knowledge regarding the structure and activity of AFGPs, as well as approaches to organic synthesis of these molecules with focus on the most recent developments.

## Introduction

Antifreeze glycopeptides (AFGPs) are a class of biological antifreeze agents found in certain Arctic and Antarctic ectotherm species. These unique compounds have the ability to regulate ice nucleation and ice crystal growth, thus creating viable conditions at temperatures below the freezing point of body fluids. Antifreeze glycopeptides typically consist of 4–55 tripeptide units of Ala–Ala–Thr that are *O*-glycosylated at the threonine side chains. The number and type of AFGPs in the blood sera of polar fish vary with habitat temperature and depth (Fields and DeVries 2015).

AFGPs are assumed to act through an adsorption–inhibition process in which the AFGP molecule irreversibly binds to the surface of an ice crystal, resulting in a freezing point depression through the Kelvin effect. A characteristic change in the morphology from spherical to hexagonal–bipyramid-shaped crystals occurs upon binding of AFGPs to specific ice crystal surfaces. AFGPs can also inhibit ice recrystallization, an Ostwald ripening process, in which larger crystals grow at the expense of smaller ones.

There are several possible applications of AFGPs due to their unique properties. These applications include use in ice-slurries and in the food industry (frozen food products, ice cream, cold preservation of fruit, and berries), as well as for biomedical purposes for the long-term cryopreservation of cells and organs or tissues [e.g., a successful cryopreservation of Nili-Ravi buffalo bull sperm was reported by Qadeer et al. (2015)] and for cryosurgery. The latter two uses can be considered as the most interesting technical applications of these materials.

Applying these compounds for biomedical and industrial purposes requires significant amounts of AFGPs in a pure form and at reasonable costs. The isolation of AFGPs from natural sources is both expensive and labour intensive. Therefore, it is necessary to develop an efficient method of producing a wide range of homogeneous and native AFGPs.

The previous major reviews on this topic (Ben 2001; Harding et al. 2003) focused on the classification, evolutionary origin, and mechanism of action of AFGPs, as well as the early structure–function studies, possible applications, and synthesis of AFGPs and their analogues. A more recent publication (Bang et al. 2013) provided a comprehensive insight into the synthesis of AFGPs and their multiple derivatives along with a report on the results of application studies. The design of novel types of ice recrystallization inhibitors, including glycopeptides, is thoroughly discussed by Balcerzak et al. (2014).

This review aims to summarize the current state of knowledge together with the trends and approaches in research regarding the structure and mechanism of action of AFGPs, as well as the most recent advances in techniques for their synthesis, along with a potential application of these methods for technical purposes.

## Structure of antifreeze glycopeptides

To survive under subzero temperature conditions that are present in the harsh environment of the Arctic and Antarctic seas, many marine organisms developed an evolutionary means of survival—the expression of antifreeze agents. AFGPs prevent the uncontrolled growth of ice in vivo by artificially lowering the freezing point below that of the surrounding seawater, which is approximately −1.8 °C.

**Fig. 1:**
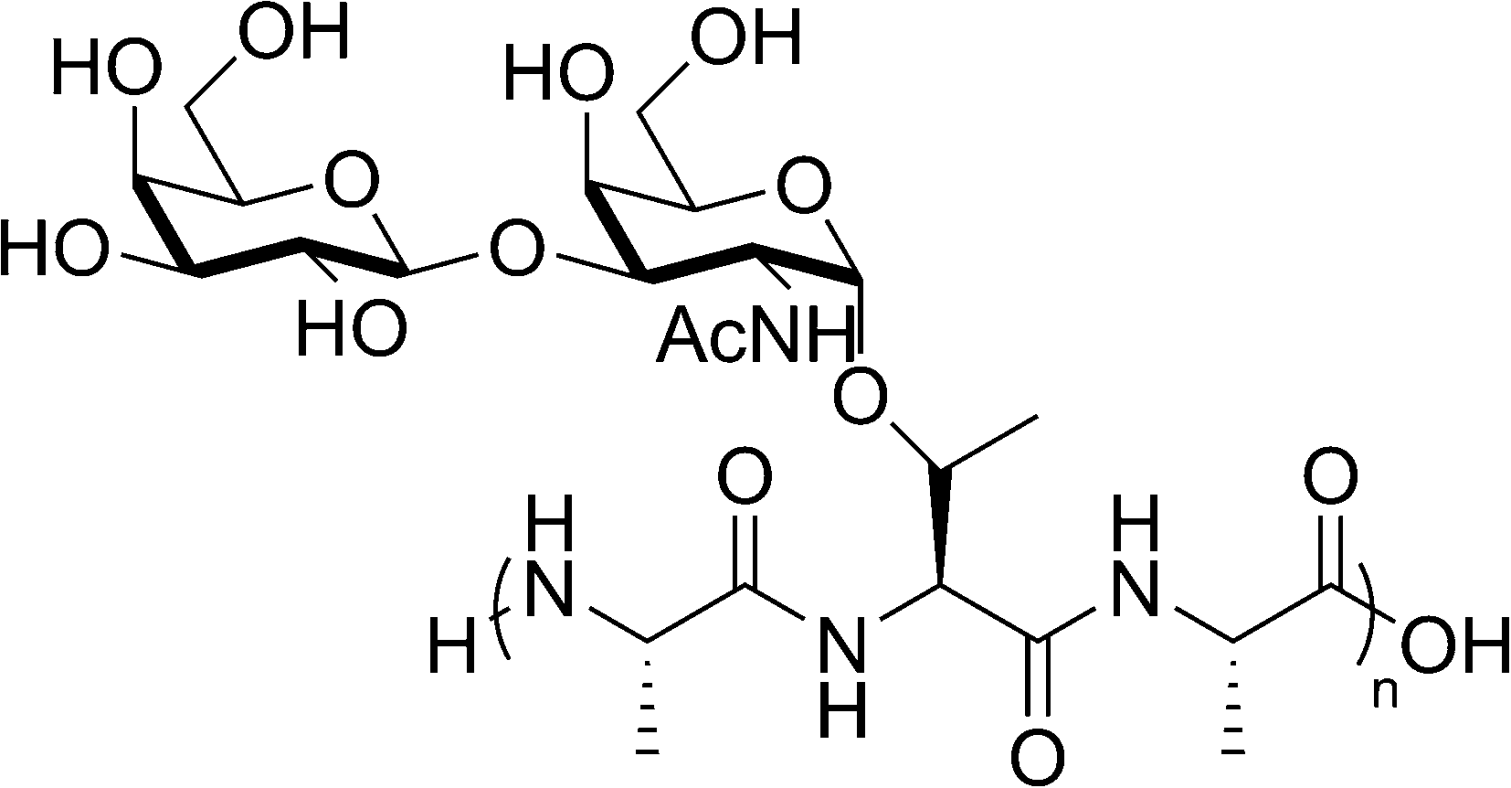
Structure of a natural AFGP (*n* = 4–55)

Antifreeze glycopeptides have been identified as the major fraction of protein in the blood sera of Arctic cod and Antarctic notothenioids. The glycopeptide group is composed of at least eight structurally related molecules that share the same function of ice crystal growth and ice recrystallization inhibition but differ regarding their peptide sequence and chain length. In a typical case, an AFGP consists of multiple repetitions of Ala–Ala–Thr units, where the threonine residue is *O*-glycosylated with the disaccharide fragment β-d-galactosyl-(1 → 3)-α-*N*-acetyl-d-galactosamine (Fig. 1) and the number of repetitions varies from 4 to 55. Following the identification of AFGPs in notothenioids, an additional division into eight subgroups (AFGP1–8) was introduced. The classification is based on their molecular weight, as shown by their relative migration rates in an electrophoretic assay (Feeney 1974). In accordance with this scheme, the AFGPs with four repetitive units and a molecular weight of 2.6 kDa are denoted as AFGP8, whereas those with the highest molecular weight of 33.7 kDa are referred to as AFGP1. The characteristics of AFGPs2–7 are between these arbitrary boundaries. A simplified version of this classification allows the grouping of AFGPs as small (AFGP6–8) and large (AFGP1–5) (Harding et al. 2003).

Although the amino-acid sequence of AFGPs can be generally regarded as conserved, a few variations that deviate from this scheme have been reported in the literature. In the large AFGPs of the notothenioids, it was shown that Ala is occasionally substituted by Pro in some of the repeated monomer units (Lin et al. 1972; Geoghegan et al. 1980; Hew et al. 1981). However, in some of the AFGPs isolated from Arctic and North Atlantic cods, the Thr residues are replaced by Arg (Fletcher et al. 1982; O’Grady et al. 1982; Burcham et al. 1986). This type of replacement results in a lack of glycosylation in the substituted positions. Additional studies focusing on other Antarctic fish species and their AFGP variants showed that traces of other amino acids could also be found, most notably Gly, Val, and Leu (Wöhrmann 1996). Overall, AFGPs can be regarded as a conserved group of molecules characterized by low amino-acid diversity and moderate size differences.

## Conformational studies

The conformational properties of AFGPs have been predominantly studied using circular dichroism spectroscopy (CD) and nuclear magnetic resonance spectroscopy (NMR). Recent achievements in this field include the introduction of more elaborate analytical methods, such as atomic force microscopy (AFM).

The first reports from CD measurements of AFGPs indicated an extended random coil structure. These results have been opposed by Franks and Morris (1978) who, after a series of studies, postulated that the CD spectrum of AFGPs resembles a β-structure in position, magnitude, and spectral form but shows an opposite sign, suggesting an unusual peptide conformation. Based on the NMR results, the group suggested that the disaccharide exposes the hydrophilic fragments to the solvent and that the hydrophobic groups are oriented towards the peptide main chain. Furthermore, from the analysis of the NMR spectra, they concluded that with this arrangement, the backbone might adopt a left-handed helical conformation with approximately three residues per turn, in a configuration similar to the polyproline II (PPII) helix. This finding was corroborated by Mimura et al. (1992). In their investigations, they proposed that an intramolecular hydrogen bond might exist between the NHAc group of the disaccharide and the carbonyl oxygen of threonine. This bonding would result in stabilization of the structure and would promote the formation of a threefold left-handed helix. The results of CD experiments in the vacuum-UV range conducted by Bush et al. (1981) implied that the structure is, indeed, similar to a threefold left-handed helix. The group postulated that the effect of the disaccharide on the overall spectrum can be regarded as negligible, implying that the main chain composition is the factor responsible for the AFGP conformation. In a subsequent study, Bush and Feeney (1986) introduced 1D and 2D NMR experiments to further elucidate the AFGP conformation. They performed ^1^H and ^13^C NMR experiments at variable temperatures, as well as nuclear overhauser effect (NOE) measurements. In the case of low-molecular-weight AFGPs, the results showed that the overall structure is temperature dependent. At higher temperatures, the AFGPs adopt the conformation of a flexible coil, but lowering the temperature results in the formation of a threefold left-handed PPII helix. However, AFGPs1–4 were determined to be flexible rods with low structure ordering. Rao and Bush (1987) attempted to verify the hypothesis that the threefold left-handed helix is the overall preferred conformation for AFGPs. Using NMR experiments and semi-empirical computational methods, they showed that this type of arrangement is one of the minimum energy conformations. However, they stated that this conformation might not be the global minimum conformation of AFGPs, because other spatial distribution possibilities appear to exist. In addition, their study demonstrated that the difference in activity between large and small AFGPs is most likely due to the differences in chain length rather than the conformation itself. The threefold left-handed helix model was also supported by Lane et al. (1998) who conducted NMR studies of a 14-residue-long AFGP isolated from Antarctic cod. The results were refined by molecular modelling methods and they demonstrated that fragments of the structure adopted the PPII conformation.

The effect of a supercooled environment on the conformational space of AFGPs was the subject of a study by Tsvetkova et al. (2002). Using solid-state NMR experiments in the presence of ice, supplemented by Fourier transform infrared spectroscopy (FTIR), they demonstrated that AFGPs undergo considerable conformational changes in response to environmental changes. They showed that in the liquid state, a large number of possible conformations exist for AFGPs, regardless of their molecular weight, with slight conformational preferences for particular AFGP subgroups. When the supercooled state is reached in the presence of ice, the degree of molecular ordering of the AFGPs is greatly increased, which is reflected by a high percentage of β turns. These results show that a certain temperature and the presence of ice might be necessary for AFGPs to adopt their functional conformation. In addition, the results show that the conformation of AFGPs must be determined under appropriate conditions to elucidate the structural–functional relationship of this group of molecules.

Additional information on the behaviour of AFGPs was provided by the data of the group led by Lavalle et al. (2000). The group studied the adsorption of AFGPs on the hydrophilic silicate surfaces of silica–titania and muscovite–silica. On the muscovite–silica surface, the glycopeptides adsorbed randomly with dimensions that were directly related to those of the individual molecules. In the case of the silica–titania surface, the initial individual deposition was followed by aggregation of the AFGPs. These results suggest that AFGPs are relatively hydrophilic, which underscores the importance of the disaccharide moieties, because the majority of the molecule is composed of the strictly hydrophobic core.

To get a better understanding of the solution conformation of AFGPs and the antifreeze activity, Bouvet et al. (2006) analysed AFGP8 in aqueous solutions using CD spectroscopy and dynamic light scattering (DLS). They demonstrated that AFGP8 forms discrete aggregates formed predominantly of dimers at concentrations above 20 mM. The size of the aggregates increased as a function of time. Results from CD spectroscopy indicated that the preferred conformation was that of a random coil. However, α-helical and β-sheet structures were found at room temperature and higher concentrations, which implies that the glycopeptides are highly flexible in solution. Bouvet et al. observed that the depression of the freezing point generated by the aggregated solution was much more effective than for monomeric AFGP8 species and suggested the importance of the cooperative mechanism in relation to the antifreeze activity. The report was the first to demonstrate the propensity for aggregation of lower molecular mass AFGPs at higher concentrations.

Further research on aggregation of AFGP8 was conducted by Younes-Metzler et al. (2007), who examined the surface patterning of AFGP8 by the solvent evaporation method on a mica surface. They demonstrated that a grid-like structure with periodic lines was obtained by the removal of the solvent from dilute protein solutions. The average aggregate height suggested the formation of a monolayer. These observations indicate that at low concentrations, AFGP8 exists as single molecules in solution. Finally, it should be stated that this result implies the existence of a 2D aggregate, in contrast to the 3D aggregation, that was shown to occur while using higher peptide concentrations.

## Mechanism of action

**Fig. 2:**
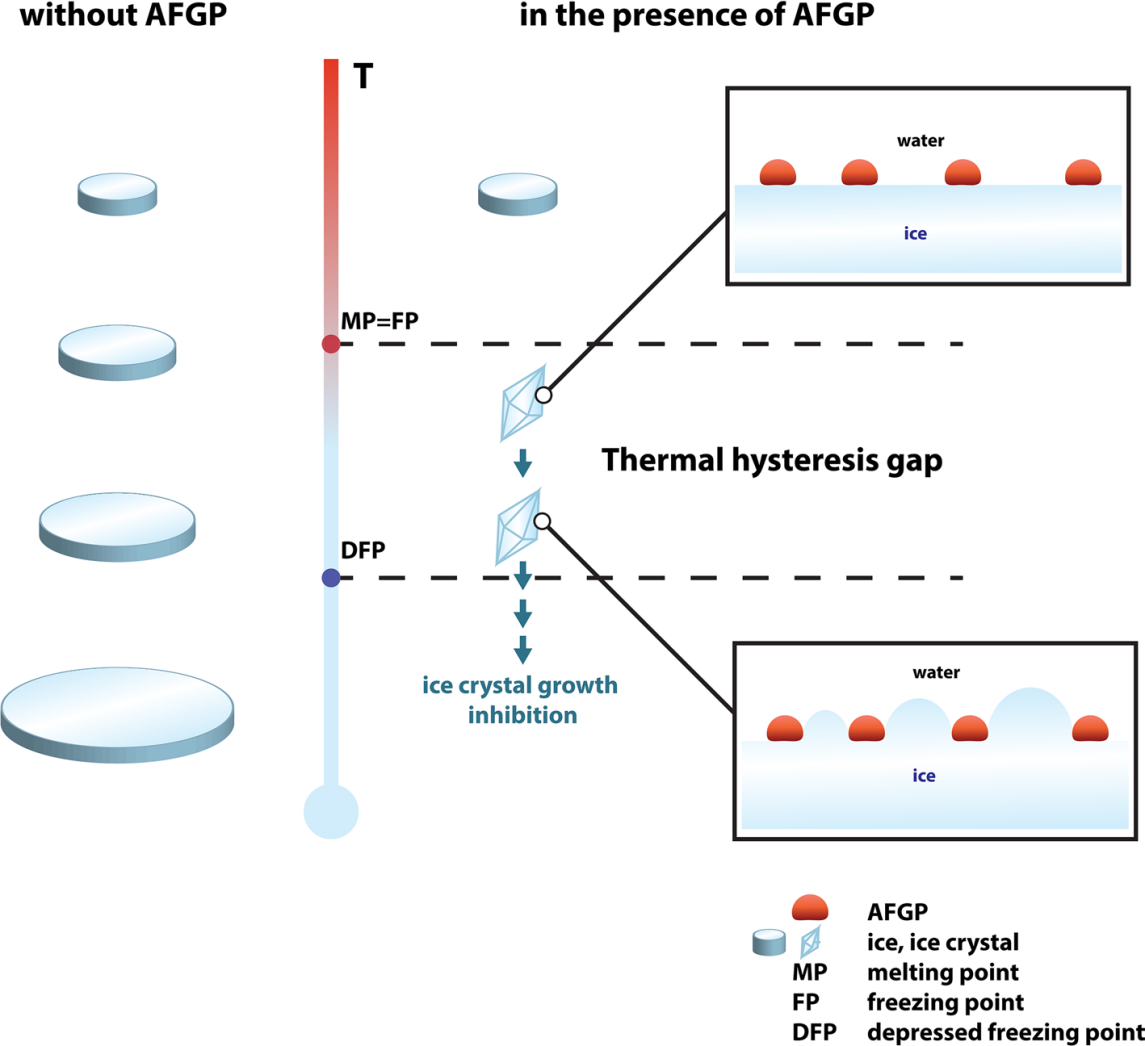
Thermal hysteresis gap is the result of the Kelvin effect caused by the introduction of AFGPs to the solution

On a purely macroscopic level, AFGPs effectively bind to the ice crystal surface (Ananthanarayanan 1989; Wilson 1993). However, the molecular mechanism responsible for this phenomenon is still poorly understood and is the topic of an ongoing debate. However, the mechanism is based on the adsorption of the peptide molecules onto the ice surface (Feeney et al. 1991; Brown et al. 1985). The binding of AFGPs onto the ice surface causes the crystals to grow in the free space between the adsorbed peptide molecules, which introduces a curvature to the ice crystal surface. After a certain amount of time, this system reaches a state in which the addition of water molecules to the convex surface is no longer energetically favourable. At this point, ice crystal growth is hindered, and a non-equilibrium freezing point depression is observed. Importantly, the melting point remains constant, while the freezing point decreases. This behaviour is the result of the surface tension effect (Kelvin effect), which states that surface curvature limits the amount of bonding that can occur between any given water molecule on the surface and its neighbours. The observed difference between the melting and freezing points is referred to as thermal hysteresis (Fig. 2). The ability to effectively generate thermal hysteresis (TH) is the crucial feature that enables AFGPs to exhibit antifreeze activity and is sometimes referred to as TH activity. In addition, AFGPs are characterized by the ability to inhibit ice recrystallization (IRI activity). Ice recrystallization is an Ostwald ripening process in which larger crystals grow at the expense of smaller ones. During recrystallization, the overall number of ice crystals is reduced and the mean size of ice crystals increases, while the total amount of ice remains constant. The rate of this process depends predominantly upon the diffusion constant of water between two neighbouring water molecules; however, in aqueous solutions containing AFGPs, the rate of ice recrystallization is reduced and can be correlated with the concentration of the antifreeze molecules (Budke et al. 2014).

**Fig. 3:**
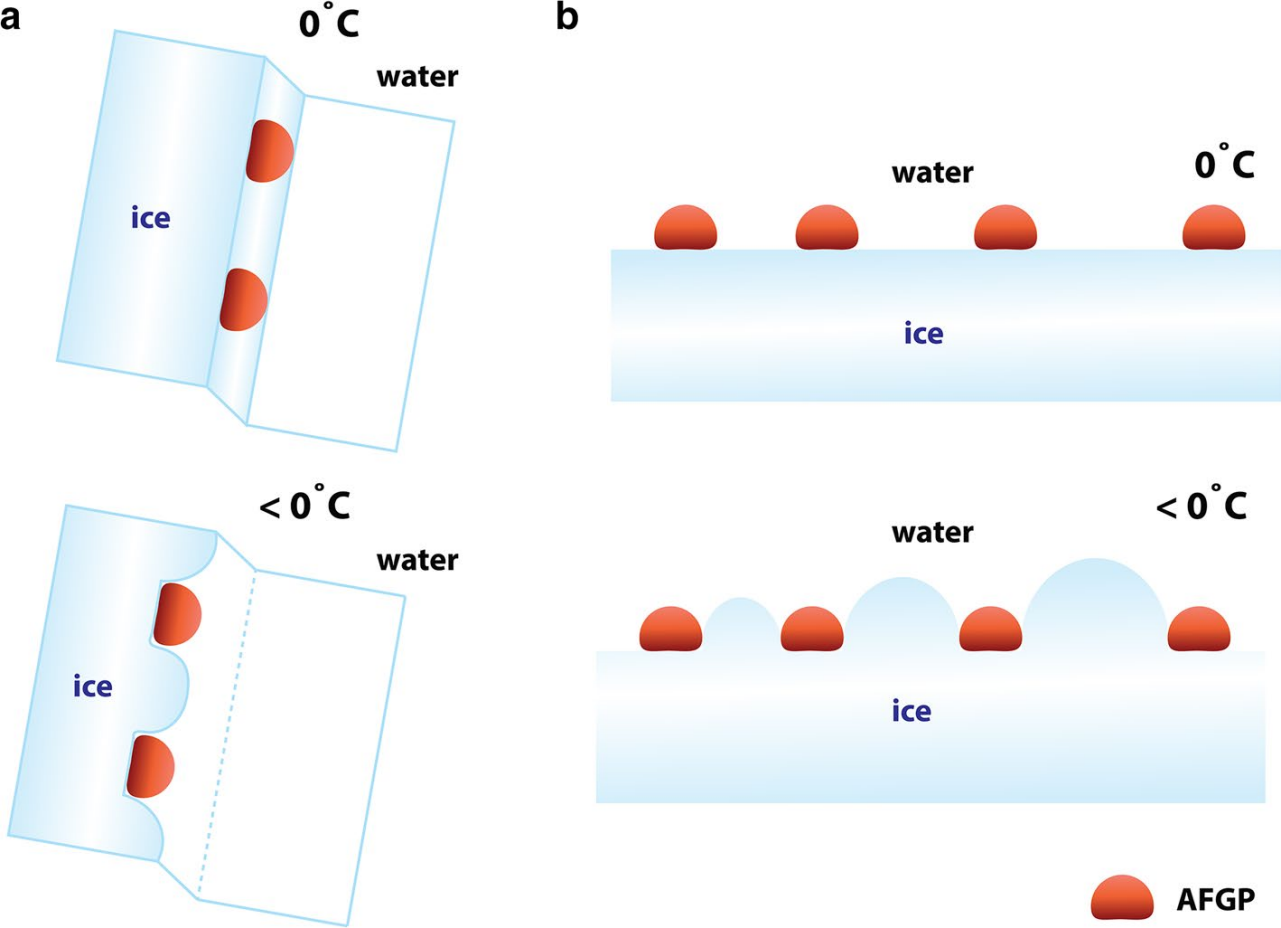
Hypothetical mechanisms of AFGP ice growth inhibition: the step-pinning model (**a**) and the mattress model (**b**)

Two hypothetical models of AFGP binding have widely been discussed in the literature. Both of the models postulate that the process of peptide adsorption onto the ice surface is irreversible, but they differ regarding the definition of the specific crystal surfaces for AFGP binding. The first model is referred to as the step-pinning model or the 3D model (Fig. 3a) and was introduced by Raymond and DeVries (1977). In this model, the AFGPs inhibit the growth of an ice step by pinning themselves to the ice surface. The disadvantage of this model is that it assumes that the growth of the ice crystal occurs in steps that advance across the plane, onto which the AFGPs are adsorbed. The second model, which provides 2D view (Fig. 3b), was proposed by Knight et al. (1991) and is named the mattress model. This model states that the AFGPs hinder the ice crystal growth perpendicular to the ice surface. The AFGP molecules adsorbed on the crystal surfaces resemble the pins of a mattress.

The irreversible nature of AFGP binding is still not well understood, but it suggests that high levels of adsorption should be observed at lower concentrations; however, there are no reports to demonstrate this behaviour (Hew and Yang 1992). In addition, it was shown that the free energy of adsorption is close to zero, which is unnatural, because for irreversible binding, large, negative values are expected (Hall and Lips 1999). These observations led to the formulation of several other hypotheses for the binding mechanism (Hall and Lips 1999; Osuga et al. 1978, 1980; Wen and Laursen 1992). However, the general principle of an irreversible adsorption-based mechanism remains the same among all of the hypotheses.

**Fig. 4:**
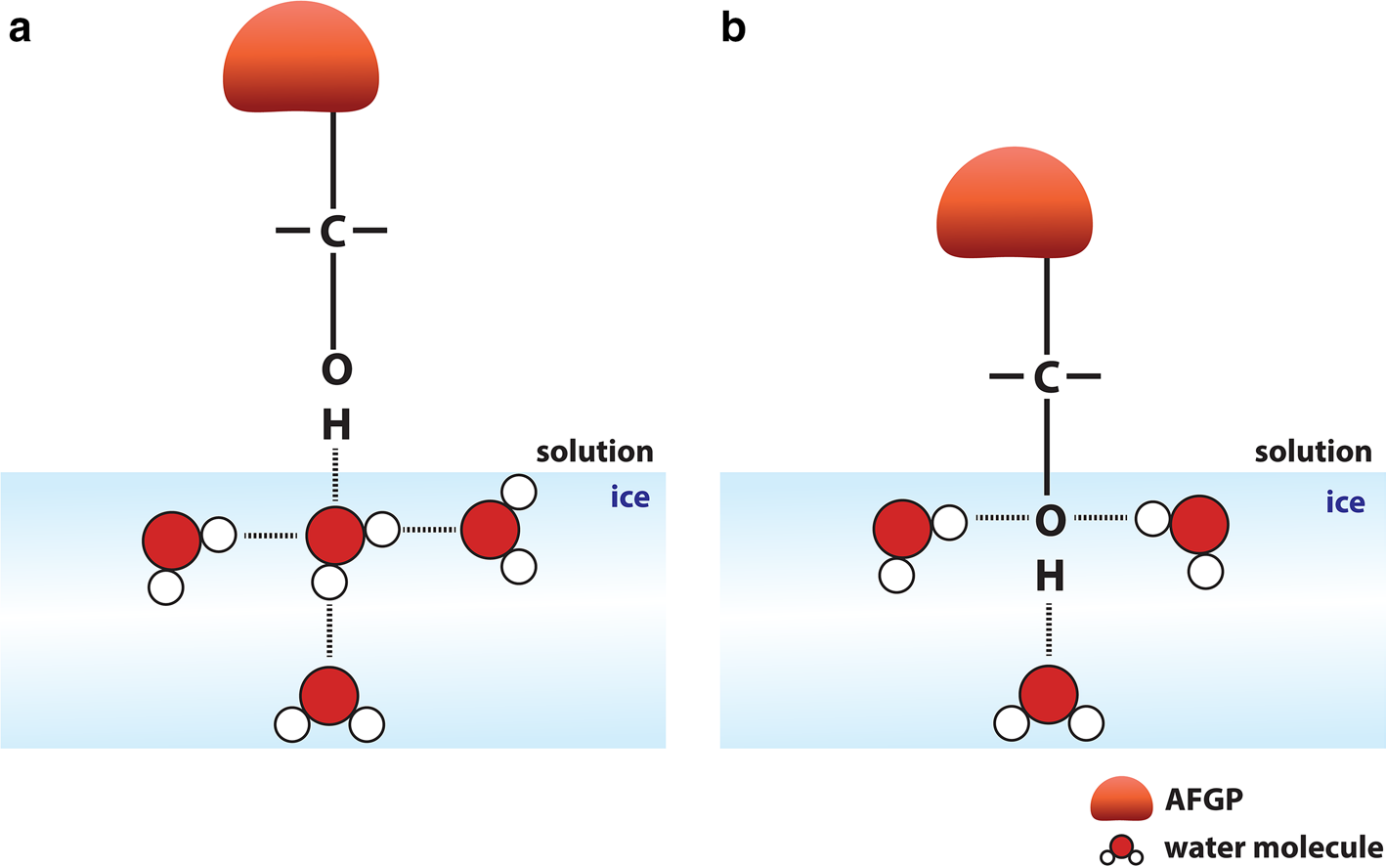
Hypothetical possibilities for the formation of hydrogen bonds between the AFGPs and the ice crystal lattice

Another subject that has extensively been analysed with respect to the binding mechanism of AFGPs is hydrogen bonding. It has widely been suggested that the tight binding that can be observed between the AFGPs and the ice surface is related to the formation of multiple hydrogen bonds between the polar groups of the carbohydrate moieties of the glycopeptides and water from the ice lattice. Interestingly, after analysing the adsorption of AFGP-7 and -8 onto the ice surface, Knight et al. (1993) concluded that the probable hydrogen-binding patterns might be insufficient to enforce the efficiency of this mechanism. The initial analysis of possible binding configurations led to the conclusion that in a best-case scenario, only two hydroxyl groups in a given carbohydrate fragment can participate in hydrogen binding. This scenario indicates that AFGP8, which contains four glycosylated residues, would be capable of forming only eight hydrogen bonds with the ice crystal surface (Fig. 4a). However, this contradicts the experimental results, indicating that AFGP8 can bind to the ice surface in an irreversible manner. Knight et al. (1993) proposed an alternative mechanism that relies on the co-crystallization of the ice crystals with the AFGP molecule. Here, the hydroxyl groups of the disaccharide units are directly incorporated into the ice crystal surface, allowing for the formation of three hydrogen bonds for each of the involved hydroxyl groups (Fig. 4b). Assuming that the orientation of the AFGPs only allows for interactions between two hydroxyl groups per disaccharide unit, the total amount of hydrogen bonds formed by AFGP8 increases from 8 to 24, which may enforce the irreversible binding.

The significance of hydrogen bonding has been questioned by some researchers. Chao and coworkers postulated that the entropic and enthalpic effects, as well as the van der Waals forces, might play a more crucial role than does hydrogen bonding in the interactions between the biological antifreeze compounds and ice (Chao et al. 1997). Similar results were proposed by Haymet et al. (1998), implying that the hydrogen bonding may not be as prevalent as the role of hydrophobic effects resulting from the highly hydrophobic peptide core, which promotes the orientation of the threonine side chains in a manner, such that their side chains are projected onto the peptide surface. Other groups attempted to evaluate the relationship between the side chain flexibility and the binding of antifreeze proteins (Gronwald et al. 1996; DeLuca et al. 1998). This resulted in further complications in the elucidation of the binding mechanism, as it has been demonstrated that distinct ‘biological antifreeze compounds’ tend to bind to different ice surfaces.

Theoretical methods were also used to support the elucidation of the AFGP mode of action. Nguyen et al. (2002) studied the behaviour of a proline-containing glycopeptide using NMR-based models and molecular dynamics. They proposed that the process of interconversion between the multiple minimum structures of the antifreeze glycopeptides might act as a thermal reservoir that hinders the growth rate of the ice crystal in a local manner. However, the theoretical simulations were performed at standard temperature (300 K), which does not provide a complete overview of the behaviour of antifreeze glycopeptides in supercooled water.

## Structure–activity relationship

The determination of the structure–activity relationship of AFGPs has been a major topic of different studies. As previously mentioned, the length of the glycopeptide chain plays a crucial role in the activity of AFGPs. Feeney and Yeh (1978) obtained AFGPs of different lengths by the subsequent subtilisin hydrolysis of larger molecules. They reported that small glycopeptides do not exhibit antifreeze activity. This remains true even for hexa- and heptapeptides which already contain two glycosylated residues, and is reflected in the fact that AFGP8 retains only ca. 30% of the activity of AFGP1.

DeVries et al. extensively analysed the effect of AFGP length on one of the characteristic properties of biological antifreeze compounds—thermal hysteresis (Knight et al. 1984). Thermal hysteresis is the depression of the freezing point and can be determined by measuring the kinetic ice growth point and then subtracting the equilibrium melting point of the solution. The highest values of thermal hysteresis were observed for larger molecules (AFGPs1–5), whereas smaller molecules showed significantly worse results.

Tachibana et al. (2004) evaluated the structural motifs that are critical for the antifreeze activity of AFGPs. The group synthesized AFGP analogues that are essentially identical to naturally occurring AFGPs. Based on the structural results from CD and NMR spectroscopy as well as activity studies, they concluded that the antifreeze activity of AFGPs strongly depends on the presence of a specific-ordered helix similar to the PPII helix. Furthermore, they showed that a polymeric AFGP requires three key motifs: an *N*-acetyl group at the C2 position of the reducing hexosamines, a γ-methyl group of the threonine residue, and an α-configuration of the *O*-glycosidic linkages between the sugars and polypeptide chain. Finally, their results indicated that the antifreeze function is preserved even in the case of dimers.

Gibson et al. (2009) synthesized a series of structurally diverse AFGP analogues containing either a peptide or vinyl backbone to test their activity for ice growth recrystallization and to determine the parts of the AFGP that contribute to the antifreeze activity. Their study showed that only the polymers that contain a hydroxyl group in the side chain could inhibit ice growth. Moreover, they demonstrated that changes might be introduced both to the backbone and to the disaccharide moieties while retaining the ice recrystallization function. This is a significant change to the previously well-founded concept, which stated that changes in the native structure result in a loss of activity.

Budke et al. (2009) reported the analysis of the Ostwald ripening of polycrystalline ice in aqueous sucrose solutions. Using the theory of Lifshitz, Slyozov, and Wagner (LSW), the kinetics of ice recrystallization was analysed at temperatures between −6 and −10 °C, and the diffusion-limited rate constant of ice recrystallization was determined. By introducing modifications to the original LSW theory to account for inhibitory effects, Budke et al. quantitatively evaluated the efficiency of ice recrystallization using small synthetic AFGPs. The sensitivity was at least two orders of magnitude greater than that of other typical methods for thermal hysteresis measurements.

The same group (Budke et al. 2014) recently evaluated the IRI efficacy for a group of 39 compounds, including antifreeze proteins (AFPs), AFGPs, and AFGP analogues. They demonstrated that native AFGPs are the most effective IRI agents, whereas the antifreeze activity is strongly reduced for monosaccharide AFGP analogues and AFGP analogues with acetyl-protected monosaccharide moieties. Furthermore, they tested the effect of different amino-acid substitutions in the peptide backbone of acetylated AFGP analogues. The results proved that a single exchange of an alanine unit into proline gives a slight increase in the activity of the glycopeptides, while replacement of two alanines by two prolines yields a complete loss of functionality. However, a modification of one of the prolines into a serine or glycine provides a slight recovery of the IRI activity. Noteworthy, AFGP diastereomers were devoid of any activity (Nagel et al. 2012a).

The results by Budke et al. mentioned above were confirmed by the recent work of Olijve et al. (2016). They reported the analysis of the IRI activity of AFGP1–5 using the circle Hough transform (CHT) algorithm. Evaluation of data from experiments performed over a wide range of concentrations demonstrated that ice crystallization kinetics is drastically reduced at concentrations higher than 0.01 μM. The value for effective inhibitory concentration obtained using the CHT procedure was equal to 0.001 μM.

The synthesis of C-linked galactosyl serine AFGP analogues as potential inhibitors of ice recrystallization was reported by Liu and Ben (2005). During the study, olefin cross-metathesis and catalytic asymmetric hydrogenation were utilized to produce divergent AFGP analogues characterized by different distances between the carbohydrate moieties and the peptide backbone. The group demonstrated a correlation between the aforementioned distance and the IRI activity. Increasing the length of the side chain resulted in lower IRI activity, whereas the most potent inhibitor was the analogue with the shortest distance between the carbohydrate fragment and the peptide backbone. They indicated that it leads to the assumption that rational design of potent recrystallization inhibitors for medical, industrial, and commercial applications is a highly valuable possibility. Further in vitro studies (Liu et al. 2007) support this claim as they demonstrated that a functional C-linked AFGP analogue possessing IRI activity and lacking thermal hysteresis is not cytotoxic to human liver cells in contrast to AFGP8. The analysed analogue hindered the activity of caspases-3 and -7, which indicated its potential effectiveness in preventing cold-induced apoptosis.

In 2013, the relationship between the structure and IRI activity of a *C*-linked AFGP analogue was analysed by the group led by (Trant et al. 2013). They demonstrated that IRI activity decreases rapidly in conjunction with lower peptide length. A minimum of three tripeptide repeats was required to observed high IRI activity at concentration of 22 mM, whereas the addition of another tripeptide unit yielded potent inhibition at 5.5 μM concentration. The results highlighted the importance of the influence of the peptide backbone and the number of tripeptide repeats on IRI activity. Furthermore, they proved that conjugation of various long alkyl chains to the original molecule resulted in increased IRI activity at low concentrations. The observation indicates that there is a realistic possibility of development of very potent small molecule inhibitors of ice recrystallization.

Czechura et al. (2008) reported the synthesis of *C*-linked AFGP analogues incorporating d-galactose, d-glucose, d-mannose, and d-talose to investigate the relationship between carbohydrate configuration and IRI activity. The results of the research indicated that the compatibility of the carbohydrate moiety with the three-dimensional hydrogen-bonded network of bulk water is inversely proportional to IRI activity. Furthermore, they provided evidence that the configuration of the hexose in *C*-linked AFGP analogues is of pivotal importance in terms of modulation of IRI activity. Their results suggested that the compatibility of the carbohydrate moiety with the hydrogen-bonded network of water is related to the hydration for each *C*-linked pyranose. Altering the levels of hydration leads to changes in the energy required to transfer a water molecule to the ice lattice and, in consequence, can result in inhibition of ice growth.

The relationship between hydration and IRI activity of *C*-linked AFGP analogues was further investigated by Tam et al. (2009) using NMR spectroscopy and molecular dynamics simulations. In one of the studied cases of a *C*-linked AFGP analogue, they observed formation of a hydrophobic ‘pocket’ between the carbohydrate moiety and the backbone. This orientation of the carbohydrate proved to be highly favourable in terms of interactions with the quasi-liquid layer of the ice lattice and most likely influenced the hydration shell of the glycopeptide, which lead to potent IRI activity.

A report on the influence of hydration dynamics on the inhibition mechanism of AFGPs was contributed by Ebbinghaus et al. (2010). Following the results of mutagenesis experiments that implied a diminished role of hydrogen bonding as the main factor in ice growth inhibition, they analysed the action of AFGPs using terahertz spectroscopy. Ebbinghaus et al. showed that the antifreeze activity is directly related to long-range collective hydration dynamics. In this model, the AFGPs act as ‘carriers’ for carbohydrates to maximize their hydration shell. The water from the hydration shell possesses a depressed freezing point compared with the bulk; consequently, freezing is not promoted.

The previous findings spurred further investigations of the hydration shell of biological antifreeze compounds. Terahertz spectroscopy and molecular dynamics simulations are powerful tools in this process. A comprehensive article describing the perspectives of the usage of these methods in the AFGP field was recently submitted by Conti Nibali and Havenith (2014). The authors highlight that recent advances in the field of THz spectroscopy allow for a better understanding of the role of hydration water in the proximity of protein surfaces in terms of its impact on structure, stability, and protein dynamics. Based on data obtained from reports on antifreeze proteins and AFGPs, the authors ascertained that THz absorption is a sensitive tool that can be used to probe minuscule changes in hydration dynamics caused by a solute. Furthermore, they stated that dynamic coupling of the THz dynamics of biomolecules along with those of their hydration shell might lead to a better understanding of the underlying biomolecular mechanisms. They proposed a ‘two-tier’ model in which the mode of action is the result of short- and long-range interactions (solute–solvent interactions and heterogeneous hydration dynamics toward functional sites). Finally, they mentioned the pioneer kinetic THz absorption (KITA) studies, as a promising direction for future research in this field.

Along this line, Krishnamoorthy et al. (2014) studied the local water dynamics around an AFGP in the presence of osmolytes using atomistic molecular dynamics simulations. By analysing the water’s hydrogen-bonding characteristics and the dipolar relaxation times, they showed a strong hindrance of the water dynamics around the AFGP molecule. Their numerical results confirmed the results obtained by Budke et al., and indicated the importance of polar units, such as the disaccharide moieties and threonine, on the ice growth inhibition process. Furthermore, they reported a considerable change of the hydration dynamics in the presence of osmolytes, such as hydroxyectoine and urea.

In another study, Mallajosyula et al. (2014) evaluated the long-range water dynamics in the presence of AFGP8 and a synthetic AFGP4. They revealed a close interplay between the glycopeptide geometry and the antifreeze properties. They demonstrated that the activity of AFGPs is closely connected to the modulation of the hydrogen-bond network of the surrounding solvent, which was demonstrated by the analysis of radial distribution functions, tetrahedral order parameters, and water–water hydrogen-bond autocorrelation functions. Mallajosyula showed that the formation of water bridges on the surface of the saccharide units affects the local tetrahedral order of water molecules in the first solvation shell. This effect propagates to the remaining solvation shells at low temperatures but appears to have significantly less effect over higher temperature ranges. This finding suggests that the long-range effect commonly occurs in an ordered water environment, which further supports the inhibition mechanism of AFGPs at low temperatures.

Another factor that has been addressed in the structure–activity relationship studies of AFGPs is their affinity towards different ice surfaces. Raymond et al. (1989) studied the binding preferences of AFGPs of divergent sizes. They concluded that in the presence of smaller AFGPs, the ice growth preferentially occurs around the c-axis and the edges of the basal plane. In contrast, when larger AFGPs were introduced, the ice crystals formed hexagonal pits on the basal plane. Further studies conducted by Knight and DeVries (1994) aimed to determine the preferences of particular AFGPs towards certain primary prism planes of the ice surface in correspondence to both the AFGP length and the concentration. Using hemisphere etching, the group demonstrated that at high concentrations, smaller AFGPs accumulate at the (1 0 $\bar{1}$ 1) plane. This preference can be shifted by lowering the concentration, which results in an accumulation of the AFGPs on the (4 1 $\bar{5}$ 0) plane. Larger AFGPs tend to accumulate at the (1 0 $\bar{1}$ 1) plane, regardless of the concentration.

## Synthesis of native AFGPs and AFGP analogues

Tsuda and Nishimura (1996) were the first to synthesize the natural tripeptide building block with the disaccharide β-d-galactosyl-(1 → 3)-α-d-*N*-acetylgalactosamine. In the first step, they prepared the monomer in 17% yield from commercially available 1,3,4,6-tetra-*O*-acetyl-2-azido-2-deoxy-α/β-d-galactopyranose. Then, the solution-phase method was used to polymerize the monomer. As a result, a mixture of polymers with an estimated mass of 6000–7300 Da (*n* = 10–12) was obtained.

Later, Tachibana et al. (2002) reported an enhanced solution-phase synthesis of native AFGPs. The amino-acid building block was exchanged from Ala–Ala–Thr to Ala–Thr–Ala with the aim of reducing the steric hindrance of the sugars during the activation of the terminal carboxyl groups for the polymerization. Placing the threonine in the middle of the tripeptide sequence provided higher polymerization yields.

Peptide synthesis in solution was replaced by solid-phase peptide synthesis (SPPS) to achieve better control over the peptide sequence and length. The first solid-phase approaches to the synthesis of the AFGP analogues were reported by Filira et al. (1990) and Meldal and Jensen (1990). However, their analogues were extremely simplified forms of the natural AFGPs, lacking the terminal galactose and the *N*-acetyl groups. Tseng et al. (2001) reported the solid-phase synthesis of natural AFGPs in 2001. Initially, the glycosylated amino acid was prepared in 60% yield. Then, the glycosylated building block was incorporated into a peptide by the classic Fmoc solid-phase approach. The final product, consisting of 14 residues, was obtained in 40% yield. In the same year, Eniade and Ben (Eniade and Ben 2001) published the fully convergent solid-phase syntheses of *C*-linked AFGP analogues containing monovalent building blocks. *C*-linked mimics of AFGP ranging from 1.6 to 3.0 kDa were prepared in 26–44% yield. Eniade (Eniade et al. 2001) proposed the synthesis of divalent building blocks suitable for the synthesis of *C*-linked AFGP analogues.

## Recent approaches in the synthesis of AFGPs

The synthesis of AFGP is divided into two main parts. The first step is the preparation of a glycosylated threonine building block. In the subsequent step, different amino acids are coupled with the peptide sequence via SPPS. The preparation of a suitable building block is crucial in the synthesis of antifreeze glycopeptides. A simple and highly efficient method of synthesizing a glycosylated amino-acid block would improve the efficiency of the entire process.

**Fig. 5:**
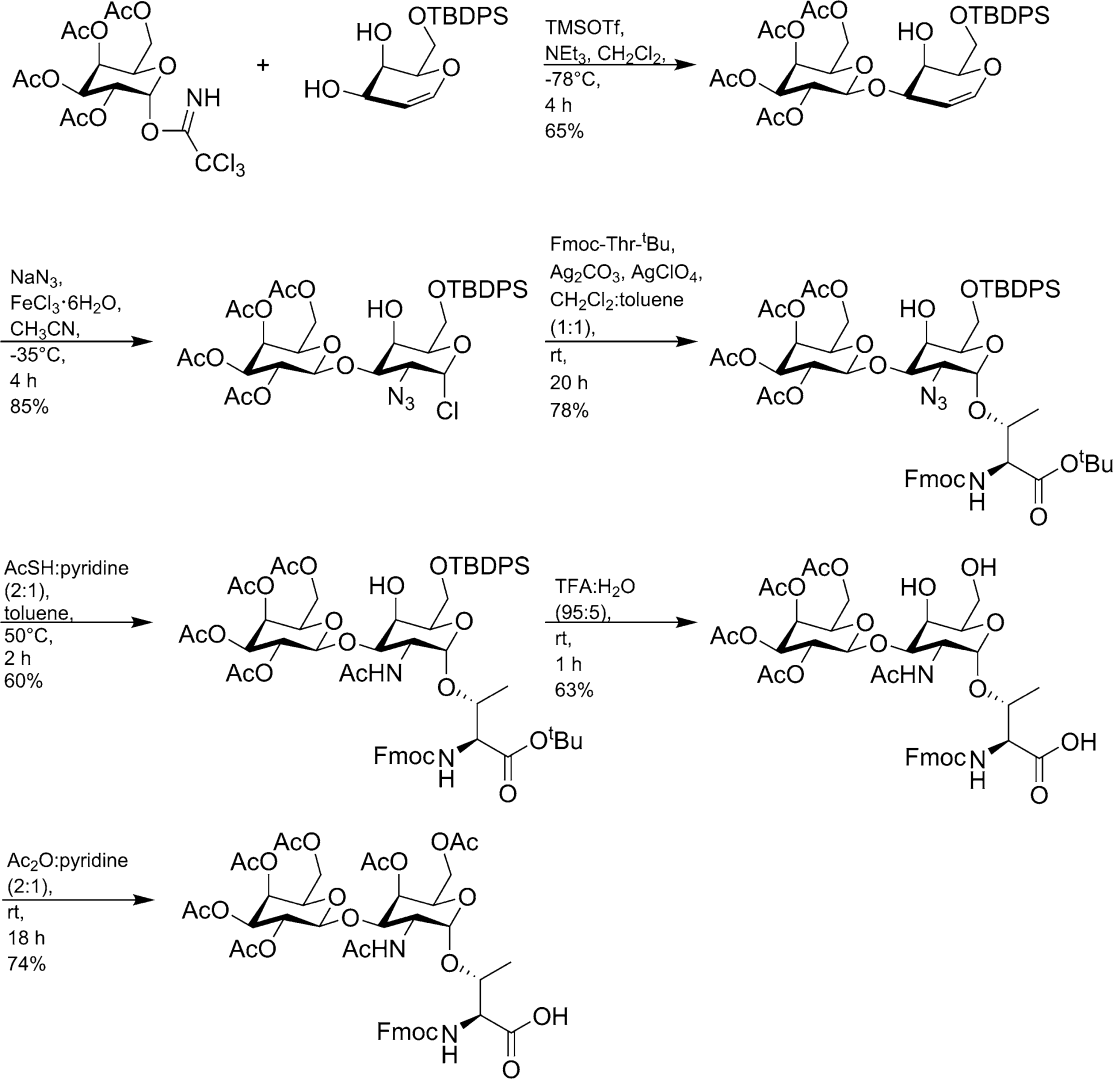
Synthesis of the protected T-antigen building block for SPPS published by Nagel et al. (2011)

Nagel et al. (2011) demonstrated a novel synthesis of the T-antigen Thr building block and its application in the microwave-assisted SPPS of AFGPs (Fig. 5). The prepared structures contained the natural T-antigen disaccharide moiety in the Ala–Ala–Thr repeating motif. The synthesis of the glycosylated building block relied on the direct azidochlorination of 3-(galactosyl)-galactal. The suggested method of synthesizing Fmoc-Thr with the disaccharide α-d-galactosyl-(1 → 3)-β-*N*-acetyl-d-galactosamine attached to the side chain required fewer synthetic steps compared with the method reported by Tseng et al. (2001), and was a selective, reliable, and scalable method of obtaining the building block.

The application of SPPS in the preparation of various glycopeptides has many advantages. It is possible to obtain relatively small glycopeptides via this method (approximately ten amino acids in a chain) due to the poor reactivity of sugar amino-acid derivatives (Izumi et al. 2013).

As a result, it might be assumed that the conventional SPPS is not suitable for the synthesis of longer AFGPs. However, the synthesis of large peptides and proteins is possible due to the native chemical ligation of small peptide derivatives used as the reactants. Recently, Wilkinson et al. conducted the convergent synthesis of homogeneous and macromolecular AFGPs from a dodecaglycopeptide intermediate using the Ala–Cys ligation followed by desulfurization. Using this strategy, it was possible to obtain homogeneous AFGP oligomers containing up to 96 amino acids in the peptide sequence with 52% yield after HPLC purification (Wilkinson et al. 2012).

**Fig. 6:**
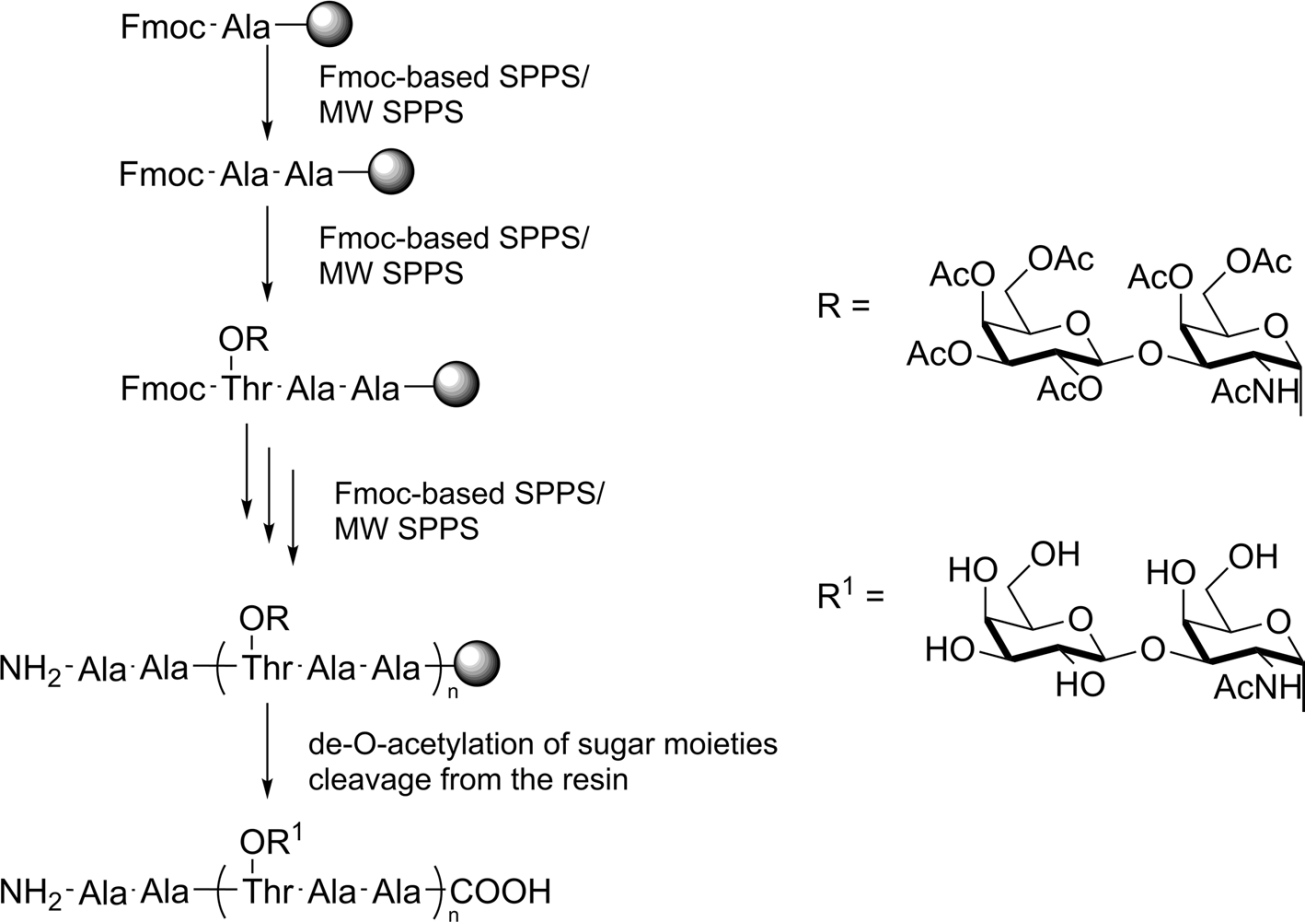
General overview of the SPPS approach for the synthesis of AFGPs; *n* = 1–5 (Izumi et al. 2013)

Another approach to improve the effectiveness of SPPS is the usage of microwave irradiation. This synthetic technique enables the rapid and large-scale synthesis of native AFGPs and their analogues. Microwave-enhanced solid-phase peptide synthesis (MW SPPS) is a simple and efficient method of preparing mono-disperse antifreeze glycopeptide oligomers. The procedure remains the same as that used for normal SPPS and consists of two steps. A schematic view for the preparation of the antifreeze glycopeptides using SPPS and MW SPPS is presented in Fig. 6. This approach enables the rapid synthesis of glycopeptides consisting of 20–40 amino acids in length with good yields (Izumi et al. 2013).

The best results in semi-automated peptide synthesis were achieved with an optimized microwave irradiation procedure with a maximum temperature of 40 °C and using a 2-chlorotrityl resin loaded with Fmoc–Ala–OH (Nagel et al. 2012b). The preparation of AFGPs under these conditions led to a time reduction for one coupling from 3 h to 45 min (Heggemann et al. 2010). Other authors recommended a higher temperature for the process, namely, 50 °C (2.45 GHz) (Izumi et al. 2013; Matsushita et al. 2005). HATU and HOAt are typically used to provide an efficient coupling of the heavily glycosylated amino-acid building blocks.

The tripeptide repeating unit of AFGP can be considered a monomer, while the peptide coupling reagents are considered as polymerization agents. van der Wal et al. (2014) published the synthesis of neo-glycopeptide oligomers. To simplify the polymerization and to increase the compatibility with more functional groups, the orthogonal azide alkyne cycloaddition polymerization catalysed by copper (CuAAC) was used in this approach (van der Wal et al. 2014).

**Fig. 7:**
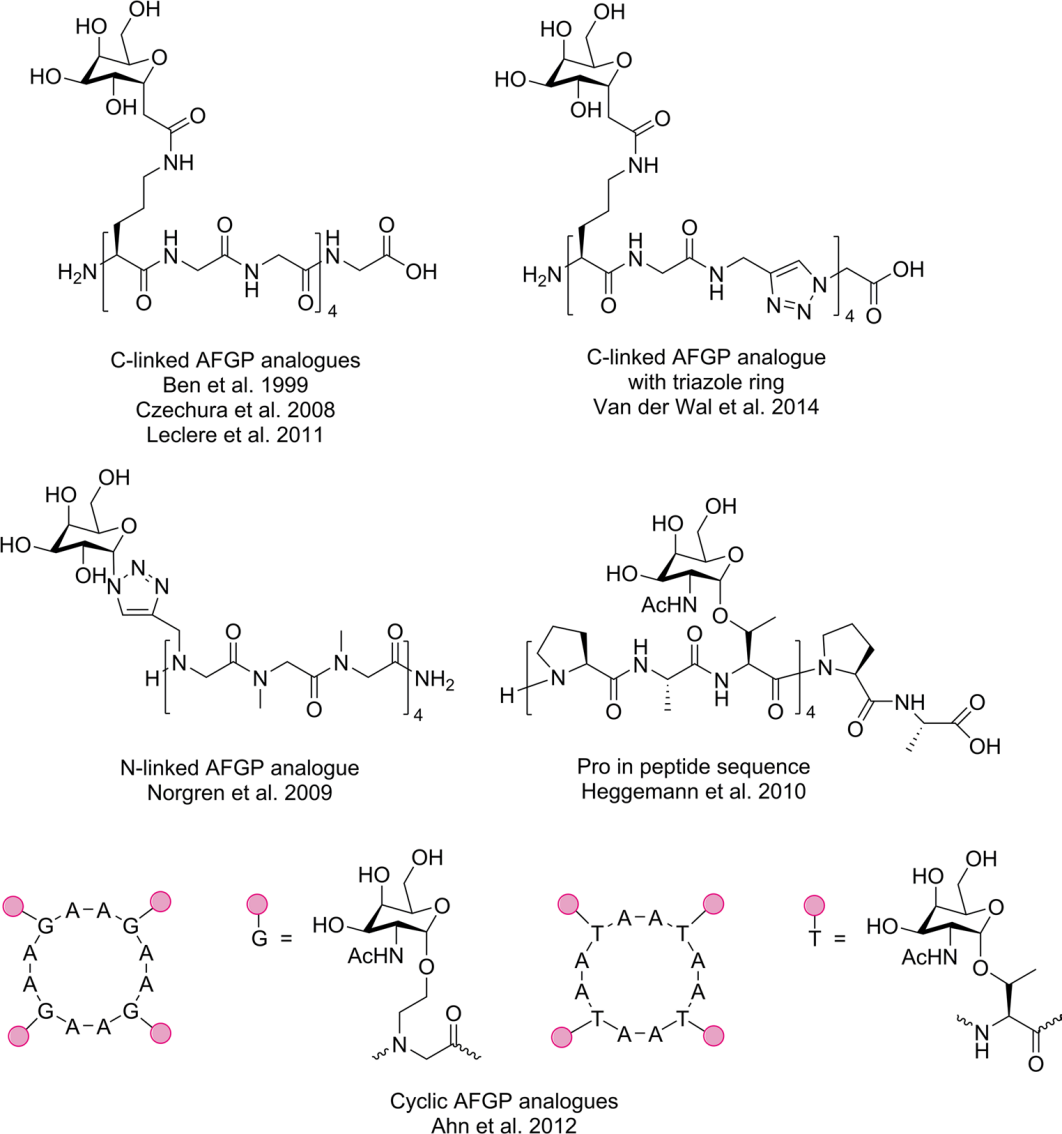
AFGP analogues synthesized by different research groups

Many studies have been dedicated to the synthetic analogues of AFGP. The derivatives may contain an *O*-linked or *N*-linked, e.g., triazole-linked, group of compounds (Miller et al. 2009; Norgren et al. 2009; Capicciotti et al. 2011; Ahn et al. 2012a, b) and *C*-linked (Ben et al. 1999; Eniade et al. 2001; Czechura et al. 2008; Tam et al. 2009; Leclère et al. 2011; Capicciotti et al. 2011), mono- and disaccharides (Fig. 7). Note that glycopeptides containing the monosaccharide *N*-acetylgalactosamine can effectively inhibit ice recrystallization, even though the monosaccharide is less structurally complex and easier to obtain (van der Wal et al. 2014).

MW SPPS is also a convenient method of preparing AFGP analogues containing sequence variations composed of Ser, Gly, and Pro residues (Heggemann et al. 2010), as well as other building blocks, such as monosaccharide—substituted Hyp (Nagel et al. 2012b). Corcilius et al. (2013) designed peptides and glycopeptides containing Hyp residues. They prepared various dodecapeptides containing Hyp residues or an Ala–Hyp–Ala repeated peptide sequence with or without α-*O*-linked *N*-acetylgalactosamine and α-*O*-linked galactosyl-β-(1 → 3)-*N*-acetylgalactosamine attached to the peptide backbone. Interestingly, the unglycosylated Ala–Hyp–Ala repeated sequence exhibits significant thermal hysteresis and the ability to change the shape of the ice crystal, which leads to the conclusion that it could bind to the ice surface (Corcilius et al. 2013).

It is also possible to obtain cyclic glycopeptides and glycopeptoids using the standard Fmoc-based solid-phase synthesis (Ahn et al. 2012a, b) or by one-pot controlled cyclization of pre-formed 6–9- and 12 peptides. The obtained cyclic compounds did not form any specific-ordered secondary structure, and surprisingly, cyclic AFGP showed the ability to inhibit the ice growth by forming hexagonal—bipyramidal ice crystals (Hachisu et al. 2009).

## Conclusions

AFGPs continue to be molecules of great interest because of their antifreeze function. However, our current state of knowledge regarding the structural preferences and mode of action of these antifreeze agents is still inconclusive. They exhibit a variety of conformational preferences, depending on the weight of the molecule as well as the temperature. The analysis of the effect of their structure on the antifreeze function requires elaborate methodologies and protocols, none of which has yielded clear results. Consequently, the determination of their mechanism of action on a molecular level still needs to be accomplished and validated. Fortunately, high-end techniques, such as AFM, might allow a further understanding of the phenomena that occur during ice growth inhibition. Furthermore, the latest advancements in computational chemistry in the fields of molecular dynamics and quantum chemical calculations offer opportunities for a complimentary, theoretical analysis of these processes. These approaches may remove the hindrances that have been present in the previous approaches and allow for the introduction of AFGPs into industrial applications.

Recent advances in the synthesis of AFGPs and their analogues indicate that microwave-assisted SPPS is an excellent tool for the preparation of heavily glycosylated peptides. The time of one coupling is reduced, and the purity and yields of the crude products are improved. Peptides composed of a various number of repeating units and different degrees of acetylation can be obtained via this method (Heggemann et al. 2010). The microwave-assisted solid-phase peptide synthesis is a universal method for the rapid and large-scale preparation of AFGPs with a precisely defined length and sequence (Izumi et al. 2013). The usage of microwave irradiation notably enhances the efficiency of all coupling reactions and improves the quality of the crude products (Matsushita et al. 2005).

However, the usage of microwave technology introduces some problems, such as racemization, the usage of expensive reagents in excess, and the necessity of maintaining optimal conditions for the synthesis (e.g., both an increase in temperature and in the time limitation of microwave irradiation during the cycles failed to accelerate the reaction). Improper reaction conditions were shown to cause massive decomposition and resulted in the peptide deglycosylation (Heggemann et al. 2010). Unfortunately, no large-scale method of synthesizing antifreeze glycopeptides exists for industrial purposes. All known advanced techniques are expensive and labour intensive, and are performed on a small scale. Another limiting factor is the usage of hazardous coupling reagents. Due to the variety of possible applications of these materials, it is vital to determine an efficient method of AFGPs synthesis. One approach could be the enzymatic glycosylation of an SPPS-prepared peptide chain. This method would mostly exclude the problematic synthesis of the Thr building block, and would reduce the usage of solvents and chemicals, placing it under the banner of green chemistry.

Note that the latest studies focus on the preparation of simplified forms of AFGPs while maintaining the antifreeze activity. The studies of simplified AFGP analogues could explain the exact mechanism of action of these compounds. Furthermore, non-toxic and structurally less complicated AFGP analogues, which can be obtained in a simpler and more efficient manner, provide an opportunity for the application of these materials in the food industry and in biomedicine.
